# Exhaled breath condensate to discriminate individuals with different smoking habits by GC–TOF/MS

**DOI:** 10.1038/s41598-017-01564-z

**Published:** 2017-05-03

**Authors:** A. Peralbo-Molina, M. Calderón-Santiago, B. Jurado-Gámez, M. D. Luque de Castro, F. Priego-Capote

**Affiliations:** 1 Department of Analytical Chemistry, Annex Marie Curie Building, Campus of Rabanales, University of Córdoba, E-14071 Córdoba, Spain; 2 Maimónides Institute of Biomedical Research (IMIBIC), Reina Sofía Hospital, University of Córdoba, E-14004 Córdoba, Spain; 3 Department of Respiratory Medicine, Reina Sofia Hospital, E-14004 Córdoba, Spain

## Abstract

Smoking is a crucial factor in respiratory diseases and lung inflammation, which are the reasons for high mortality worldwide. Despite the negative impact that tobacco consumption causes on health, few metabolomics studies have compared the composition of biofluids from smoker and non-smoker individuals. Exhaled breath condensate (EBC) is one of the biofluids less employed for clinical studies despite its non-invasive sampling and the foreseeable relationship between its composition and respiratory diseases. EBC was used in this research as clinical sample to compare three groups of individuals: current smokers (CS), former smokers (FS) and never smokers (NS). Special attention was paid to the cumulative consumption expressed as smoked pack-year. The levels of 12 metabolites found statistically significant among the three groups of individuals were discussed to find an explanation to their altered levels. Significant compounds included monoacylglycerol derivatives, terpenes and other compounds, the presence of which could be associated to the influence of smoking on the qualitative and quantitative composition of the microbiome.

## Introduction

Cigarette smoke is a source of toxicants that enter into contact with the microbiome in the respiratory tract perturbing the microbial ecology with direct incidence on immunology, oxygen deprivation or other mechanisms^1, 2^. Loss of beneficial species and appearance of new species in the respiratory tract due to smoking can lead to colonization by pathogens and ultimately to diseases. Thus, smoking is a key contributor to respiratory diseases, which are considered the most preventable cause of premature death in the United States^3^ and the major factor for development of chronic obstructive pulmonary disease (COPD) and lung cancer^4^, two diseases that cause a large part of respiratory deaths in Europe^5^.

Smoking cessation has major and immediate health benefits for individuals of all ages. Several studies have highlighted that cardiovascular risk is reduced in smokers who give up cigarettes even after a first myocardial infarction^6, 7^. For COPD patients, smoking cessation has shown to be an integral component of rehabilitation courses^4^ and also decreases the risk of different cancers, with special emphasis on lung cancer, and stroke^8^. In a recent study, Jerjes *et al*. demonstrated that reduction/cessation of chronic smoking and drinking tends to significantly reduce mortality in patients with oral cancer^9^. Moreover, other reports concluded that former smokers (FS) live longer than current smokers (CS). For example, persons who quit smoking before age 50 have one-half the risk of dying during the next 15 years compared with continuing smokers^10–12^. In this context, Boué *et al*. studied the effect of cigarette smoke exposure and the benefits of its cessation concluding that cigarette smoke exposure induced a series of metabolic as well as adaptive and innate inflammatory responses in murine respiratory tissues that were progressively deactivated by smoking cessation^13^. Nevertheless, for some cancers, especially for adenocarcinoma, the risk remains high in FS as compared with non-smokers (NS)^14, 15^.

Concerning ‘omics’ approaches, metabolomics seems to be an ideal tool to assess the impact of cigarette smoke on human exposure and health. Notwithstanding the importance of knowing how the metabolism is affected by the smoking habit, few metabolomics studies have been targeted at comparing the composition of any biofluid from CS and NS^11, 16^. In a recent study, the tobacco-related global metabolome in blood from CS and NS was identified by UPLC–QTOF^17^. Apart from identification of nicotine metabolites, there was a characteristic metabolic profile contributing to discriminate CS and NS individuals. The involved metabolites, assessed by MS/MS, showed the potential to identify metabolic phenotypes and new metabolites related to cigarette exposure and toxicity^17^.

Other relevant study on association of metabolite concentrations with smoking habit showed the reversion of metabolite variations after smoking cessation and demonstrated the results using protein–metabolite networks^11^. The study reported the association of smoking with variations in the concentration of amino acids, ether lipids and glycerophospholipid metabolism. The smoking-related changes in the profile of serum metabolites were found reversible after quitting smoking. This fact indicates the remarkable benefits of smoking cessation and provides a link to cardiovascular diseases benefits.

Contrarily to expectations, exhaled breath condensate (EBC) has been scarcely used for metabolomics analysis of the smoking habit^16^. The aim of the present research was to identify metabolites related to this habit by comparison of EBC samples from CS, FS and NS. For this purpose, metabolomic profiles of samples from CS, FS and NS individuals were obtained using the methodology developed by Peralbo-Molina *et al*. based on gas chromatography coupled to mass spectrometry in high resolution mode (GC–TOF/MS)^18^. This study has enabled the identification of compounds associated to the smoking habit, which are accumulated or not in EBC, and others that either disappear or appear after quitting the habit.

## Experimental

### Cohort selected for the study

EBC samples were collected from 119 volunteers early in the morning before breakfast, and stored at −80 °C in the biorepository of the Reina Sofía Hospital (Córdoba, Spain). All the individuals were, recruited at the Department of Respiratory Medicine according to the exclusion criteria described below. Chronic obstructive pulmonary disease (COPD) patients, diagnosed by symptomatic detection of chronic cough, expectoration and dyspnea, and confirmed by spirometry, were also discarded. The volunteers of this study were not subjected to spirometry since there are not evidences about the utility of this technique as screening tool in asymptomatic individuals^19^. Table 1 shows the main characteristics of the cohort expressed as mean value and standard deviation. The cohort included 61 individuals who had never smoked (NS), without either clinical symptoms or abnormalities in the chest radiograph. This group of individuals was aged 59 ± 8 years, and 77% of them were males. The current smokers (CS) group was formed by 32 individuals 58 ± 7 years old, and 75% of them were males. The former smokers (FS) group included 26 individuals 60 ± 8 years old −92% of them were males. Supplementary Table 1 lists the data from each individual such as age, sex, body mass index (BMI) and cigarettes consumption expressed as smoked pack-year. This parameter is frequently used to rank smoker individuals as a function of the cumulative smoking habit (0, 0–24, 24–45, >45 pack-year). The cohort included 27 individuals with normal weight (BMI < 25 kg/m^2^), 42 overweight individuals (25 < BMI < 30 kg/m^2^) and 50 obese volunteers (BMI > 30 kg/m^2^). Nevertheless, no statistical significance of age and BMI among groups was checked by Mann-Whitney test. Despite the fact that 42% of volunteers were obese, it is worth mentioning that this cohort is not far from the real situation since in 2014 the World Health Organization concluded that 39% of adults were overweight individuals, while 13% of them were obese^20^. Independence from sex was assessed by Fisher’s exact test.

**Table 1:** Generic information about each group including mean age and body mass index.

Type	Number	Sex (% male)	Age (years)	BMI (kg/m^2^)
NS	61	77.05	59 ± 8	29 ± 4
FS	26	92.31	60 ± 8	30 ± 4
CS	32	75	58 ± 7	29 ± 5

Criteria for exclusion of patients were: (a) coexistence of extrapulmonary tumoral pathology or treatment with cytostatic drugs for a different neoplasm; (b) diagnostic of neoplasm in the last five years; (c) unjustified weight loss (≥10%) during the last year; (d) severe disorders of any organ such as grade IV heart failure, advanced hepatic cirrhosis, chronic renal (stages 4–5), or lung disease not related to smoking, including interstitial pneumopathy, pneumonia, tuberculosis, etc.).

All experiments were carried out in accordance with the ethical principles of human medical research (World Medical Association, Helsinki Declaration 2004). The ethical review board of Reina Sofía Hospital (Córdoba, Spain) approved and supervised this clinical study. Individuals selected for this study were informed to obtain consent prior to sample collection.

### Procedure for EBC collection

The ECOScreen2 device (FILT Thorax-und LungenDiagnostik GmbH, Berlin, Germany) used for sampling collects and condenses the EBC in disposable polyethylene bags at −20 °C and is able to collect EBC into two separate bags for physical separation between the air contained in the upper airway and that in the distal airway^21^. This configuration makes saliva contamination highly unlikely^22^. The sampler was modified by insertion of a commercial protection filter from Scharlab (Barcelona, Spain) over the inlet air valve to avoid the entrance of exogenous organic compounds and particles from the room atmosphere. This filter was periodically changed before saturation.

In addition, some conditions were taken into account by volunteers in order to avoid contamination: (1) smokers were advised to avoid the consumption of tobacco at least 12 h prior to collection; (2) volunteers attend on an empty stomach, (3) volunteers must not use colony, makeup or lipstick the collection day; (4) volunteers do not have to perform forced expiratory maneuvers prior to collection.

Tidal breathing and a nose-clip were used for 15 min, time required to collect an average EBC volume of 1.5 mL from the distal airway. The samples were divided into 100 µL aliquots and the vials stored at −80 °C until analysis. All samples were analyzed within 3 months after collection.

### Reagents

Hexane *Trace*SELECT® grade from Sigma–Aldrich (St. Louis, USA) was used for sample preparation. An alkane standard mixture (from C10 to C40), also from Sigma–Aldrich, was tested by GC–MS analysis to establish the retention index (RI) calibration. Deionized water (18 mΩ · cm) provided by a Milli-Q water purification system from Millipore (Darmstadt, Alemania) was also used.

### Instruments and apparatus

Homogenization of the extracts was carried out by an MS2 Vortex (IKA, Germany). An Agilent 7890 A Series GC system coupled to an Agilent 7200 UHD Accurate-Mass QTOF hybrid mass spectrometer equipped with an electron impact (EI) source (Santa Clara, CA, USA) was used to analyze EBC samples. The analytical sample was monitored in high resolution mode.

### Sample preparation

Sample preparation consisted of liquid–liquid extraction using hexane as extractant. In all cases, 100 µL aliquots of EBC and 100 µL hexane were vortexed in a glass insert at room temperature for 1 min. Then, the organic phase was isolated and put into a new glass insert for analysis. To eliminate exogenous interferents, blanks prepared with water were analyzed as the samples.

### GC–TOF/MS analysis

GC–TOF/MS analyses were performed by electron impact ionization (EI) mode at 70 eV and controlled by MassHunter Acquisition B.06. The GC separation was carried out by a fused silica DB-5MS-UI 30 m × 0.25 mm i.d. 0.25 μm film thickness capillary column. The temperature program of the GC oven started at 60 °C (1/min held), followed by a temperature ramp of 10 °C/min to final 300 °C (2 min held). Post-run time was programmed for 4 min up to 310 °C to assure complete elution of the injected sample. Pulsed splitless injections of 1 μL sample were carried out at 250 °C, and ultrapure grade helium was used as the carrier gas at 1.0 mL/min. The interface and ion source were set at 280 and 300 °C, respectively. A solvent delay of 5.5 min was used to prevent damage in the ion source filament. The TOF detector was operated at 5 spectra/s in the mass range *m*/*z* 50–550 and the resolution was 8500 (full width half maximum, FWHM) at *m*/*z* 501.9706. Mass-spectrometric grade PFTBA (perfluorotri-*n*-butylamine) was used for daily mass calibration. Tentative identification of metabolites was performed by searching MS spectra on the NIST 11 database taking into account the RI values.

### Data processing and statistical analysis

Unknown Analysis software (version 7.0, Agilent Technologies, Santa Clara, CA, USA) was used to process all data obtained by GC–TOF/MS in full scan mode. Treatment of raw data files started by deconvolution of potential molecular features (MFs) with the suited algorithm included in the software. For this purpose, the deconvolution algorithm considered all ions exceeding 1500 counts for the absolute height parameter. Additionally, the accuracy error and the window size factor were set at 50 ppm and 150 units, respectively. After extraction of MFs, data files in compound exchange format (.cef files) were created for each sample and exported into the Mass Profiler Professional (MPP) software package (version 12.1, Agilent Technologies, Santa Clara, CA, USA) for further processing.

In the next step, the data were processed by alignment of the potential MFs according to their retention time and *m*/*z* value using a tolerance window of 0.3 min and an accuracy error of 15 ppm. The MFs from the analysis of blanks were removed from the data set of MFs from the EBC samples. The extraction algorithm confirmed the efficiency of this filtering step. Stepwise reduction of the MFs number was based on frequency of occurrence by comparing repetitions of the same group of individuals. A filter by frequency was set at 100%, thus ensuring detection of each MF in all the injected replicates from all samples composing each of the three groups.

In the last step, the resulting MFs were exported (.cef file) for recursive analysis. For this purpose, the Quantitative Analysis software (version 7.0, Agilent Technologies, Santa Clara, CA, USA) was used to reintegrate all potential compounds found in the analyzed samples. The resulting table was exported in coma separated value format (.csv file) and reprocessed with the Mass Profiler Professional (MPP) software package. A filter to eliminate samples by setting within-replicates variability above 10% was applied to assure the effectiveness of the recursive analysis. Finally, the data set was normalized by logarithmic transformation of the ratio between the peak area of each MF and the total useful mass spectrometry signal (MSTUS) corresponding to all the samples^18, 23^.

The resulting data set was then subjected to supervised analysis by partial least squares discriminant analysis (PLS-DA). Cross validation by using an N-fold model was selected as validation approach. With this model, the classes in the input data are randomly divided into N equal parts; N–1 parts are used for training and the remaining part is used for testing. The process is repeated N times, with a different subset being used for testing in an iterative process. Thus, each row is used at least once in training and once in testing, and a confusion matrix is generated. The complete process can be repeated as many times as specified by the number of repetitions. Validation in this research consisted of ten repetitions and a fold number of three.

The software Statgraphics Centurion (XVI version 16.1.18, Statpoint Technologies) was used for statistical analysis to study the influence of smoking habit and smoking load on each metabolite. Finally, the platform Metaboanalyst 3.0 (http://www.metaboanalyst.ca/) was used to perform Random Forest Analysis and to obtain ROC curves for the most significant compounds^24^.

Identification was firstly carried out by searching MS spectra in the NIST11 database. Only identifications with a match factor and a reverse match factor higher than 700 were considered as valid. RI values included in the NIST database were also taken into account to support identifications. An RI calibration model was built by comparing the RI values of an alkane standard mixture (composed by alkane between C10 to C40 with an even number of carbons) with the chromatographic method used in this research and the RI values provided by the NIST database. Supplementary Figure 1 shows the RI calibration line obtained by this approach. The requirement to accept NIST identifications was that the difference between the theoretical and the experimental RI obtained by extrapolation in the calibration curve should be within ±100 units.

The NIST database does not contain high resolution MS information as provided by the TOF detector. For this reason, a third step was included to validate identification of each MF by high resolution mass spectrometry. Thus, the tentative molecular formula for the parent ion [M]+ and the most intense fragment ions obtained for each MF should fit the NIST identification by setting a cut-off value of 10 ppm in mass accuracy. Table 2 lists the 44 identified compounds classified by chemical families.

**Table 2:** Compounds in EBC identified by GC–TOF/MS analysis.

Compound name	Retention time	Formula	CAS ID	Fragments	Family
Eucalyptol	5.99	C_10_H_18_O	470-82-6	154.1361 – [C_10_H_18_O]^+^ 139.1119 – [C_9_H_15_O]^+^ 93.0695 – [C_7_H_9_]^+^	Aliphatic heteropolycyclic compounds (oxanes)
Indole	9.90	C_8_H_7_N	120-72-9	117.0558 – [C_8_H_7_N]^+^ 90.0448 [C_7_H_6_]^+^ 74.0145 – [C_6_H_2_]^+^	Aromatic heteropolycyclic compounds (indoles)
Benzoic acid 4-ethoxy-ethyl ester	12.92	C_11_H_14_O_3_	23676-09-7	194.0425 – [C_11_H_14_O_3_]^+^ 149.0581 – [C_9_H_9_O_2_]^+^ 121.0269 – [C_7_H_5_O_2_]^+^	Aromatic homomonocyclic compounds (benzene and substituted derivatives)
Benzoic acid methyl ester	6.95	C_8_H_8_O_2_	93-58-3	136.0514 – [C_8_H_8_O_2_]^+^ 105.0332 – [C_7_H_5_O]^+^ 77.0378 – [C_6_H_5_]^+^	Aromatic homomonocyclic compounds (benzoic acid derivatives)
3,5-Di-*t*-butyl-4-hydroxycinnamic acid	20.16	C_17_H_24_O_3_	22014-01-3	276.1712 – [C_17_H_24_O_3_]^+^ 261.1479 – [C_16_H_21_O_3_]^+^ 177.0896 – [C_11_H_13_O_2_]^+^	Aromatic homomonocyclic compounds (cinnamic acid derivatives)
*p*-Cresol	6.54	C_7_H_8_O	106-44-5	136.0514 – [C_8_H_8_O_2_]^+^ 105.0332 – [C_7_H_5_O]^+^ 77.0378 – [C_6_H_5_]^+^	Aromatic homomonocyclic compounds (phenols and derivatives-cresol)
Benzyl alcohol	5.96	C_7_H_8_O	100-51-6	108.0565 – [C_7_H_8_O]^+^ 91.0535 – [C_7_H_7_]^+^ 79.0533 – [C_6_H_7_]^+^	Aromatic homomonocyclic compounds (primary alcohols)
Isopropyl laurate	14.97	C_15_H_30_O_2_	10233-13-3	201.1835 – [C_12_H_25_O_2_]^+^ 157.1203 – [C_9_H_17_O_2_]^+^ 102.0656 – [C_5_H_10_O_2_]^+^	Lipids (fatty acid esters)
Palmitic acid methyl ester	17.34	C_17_H_34_O_2_	112-39-0	270.2545 – [C_17_H_34_O_2_]^+^ 227.1998 – [C_14_H_27_O_2_]^+^ 143.1048 – [C_8_H_15_O_2_]^+^	Lipids (fatty acid esters)
Oleic acid methyl ester	19.04	C_19_H_36_O_2_	112-62-9	296.2702 – [C_19_H_36_O_2_]^+^ 264.2442 – [C_18_H_32_O]^+^ 81.0685 – [C_6_H_9_]^+^	Lipids (fatty acid esters)
Stearic acid methyl ester	19.28	C_19_H_38_O_2_	112-61-8	298.2862 – [C_19_H_38_O_2_]^+^ 255.2315 – [C_16_H_31_O_2_]^+^ 87.0436 – [C_4_H_7_O_2_]^+^	Lipids (fatty acid esters)
Palmitoleic acid	17.45	C_16_H_30_O_2_	373-49-9	236.2122 – [C_16_H_28_O]^+^ 98.0710 – [C_6_H_10_O]^+^ 69.0689 – [C_5_H_9_]^+^	Lipids (fatty acids and conjugates)
Palmitic acid	17.70	C_16_H_32_O_2_	57-10-3	227.1997 – [C_14_H_27_O_2_]^+^ 129.0891 – [C_7_H_13_O_2_]^+^ 73.0279 – [C_3_H_5_O_2_]^+^	Lipids (fatty acids and conjugates)
Stearic acid	19.58	C_18_H_36_O_2_	57-11-4	284.2706 – [C_18_H_36_O_2_]^+^ 129.0908 – [C_7_H_13_O_2_]^+^ 73.0281 – [C_3_H_5_O_2_]^+^	Lipids (fatty acids and conjugates)
Glycidol stearate	22.48	C_21_H_40_O_3_	7460-84-6	297.2436 – [C_18_H_33_O_3_]^+^ 98.0719 – [C_6_H_10_O]^+^ 71.0848 – [C_5_H_11_]^+^	Lipids (fatty acids and conjugates)
Undecanol	11.25	C_11_H_24_O	112-42-5	111.1157 – [C_8_H_15_]^+^ 83.0844 – [C_6_H_11_]^+^ 69.0691 – [C_5_H_9_]^+^	Lipids (fatty alcohols)
1-Hexadecanol 2-methyl	15.99	C_17_H_36_O	2490-48-4	111.1160 – [C_8_H_15_]^+^ 97.1006 – [C_7_H_13_]^+^ 69.0691 – [C_5_H_9_]^+^	Lipids (fatty alcohols)
Oleamide	21.33	C_18_H_35_NO	301-02-0	281.2679 – [C_18_H_35_NO]^+^ 126.0914 – [C_7_H_12_NO]^+^ 72.0438 – [C_3_H_6_NO]^+^	Lipids (fatty amides)
11-Eicosenamide	23.01	C_20_H_39_NO	10436-08-5	126.0915 – [C_7_H_12_NO]^+^ 72.0439 – [C_3_H_6_NO]^+^ 309.2973 – [C_20_H_39_NO]^+^	Lipids (fatty amides)
Erucamide	24.57	C_22_H_43_NO	112-84-5	337.3338 – [C_22_H_43_NO]^+^ 126.0916 – [C_7_H_12_NO]^+^ 72.0440 – [C_3_H_6_NO]^+^	Lipids (fatty amides)
Monopalmitin	22.57	C_19_H_38_O_4_	542-44-9	299.2577 – [C_18_H_35_O_3_]^+^ 257.2462 – [C_16_H_33_O_2_]^+^ 239.2366 – [C_16_H_31_O]^+^	Lipids (glycerolipids)
Monostearin	24.14	C_21_H_42_O_4_	123-94-4	327.2897 – [C_20_H_39_O_3_]^+^ 267.2677 – [C_18_H_35_O]^+^ 98.0723 – [C_6_H_10_O]^+^	Lipids (glycerolipids)
Hedione	14.41	C_13_H_22_O_3_	24851-98-7	83.0479– [C_5_H_7_O]^+^ 97.0623– [C_6_H_9_O]^+^ 226.1566 – [C_13_H_22_O_3_]^+^	Lipids (lineolic acids and derivatives-jasmonic acids)
D-Limonene	5.94	C_10_H_16_	5989-27-5	136.1227 – [C_10_H_16_]^+^ 121.0992 – [C_9_H_13_]^+^ 79.0524 – [C_6_H_7_]^+^	Lipids (prenol lipids-monoterpenes)
Cumyl alcohol	6.81	C_10_H_14_O	617-94-7	121.0644 – [C_8_H_9_O]^+^ 103.0530 – [C_8_H_7_]^+^ 91.0537 – [C_7_H_7_]^+^	Lipids (prenol lipids-monoterpenes)
Linalol	6.99	C_10_H_18_O	78-70-6	136.1218 – [C_10_H_16_]^+^ 93.0679 – [C_7_H_9_]^+^ 71.0844 – [C_5_H_4_]^+^	Lipids (prenol lipids-monoterpenes)
Camphor	7.84	C_10_H_16_O	464-48-2	152.1192 – [C_10_H_16_]^+^ 137.0963 – [C_9_H_13_O]^+^ 95.0852 – [C_7_H_11_]	Lipids (prenol lipids-monoterpenes)
Camphol	8.16	C_10_H_18_O	507-70-0	121.1000 – [C_9_H_13_]^+^ 95.0853 – [C_7_H_11_]^+^ 77.0381 – [C_6_H_5_]^+^	Lipids (prenol lipids-monoterpenes)
Levomenthol	8.25	C_10_H_20_O	2216-51-5	138.1379 – [C_10_H_18_]^+^ 95.0837 – [C_7_H_11_]^+^ 81.0683 – [C_6_H_9_]^+^	Lipids (prenol lipids-monoterpenes)
Terpineol	8.52	C_10_H_18_O	98-55-5	136.1244 – [C_10_H_16_]^+^ 121.1007 – [C_9_H_13_]^+^ 93.0695 – [C_7_H_9_]^+^	Lipids (prenol lipids-monoterpenes)
Squalene	24.84	C_30_H_50_	111-02-4	410.3907 – [C_30_H_50_]^+^ 121.0994 – [C_9_H_13_]^+^ 81.0686 – [C_6_H_9_]^+^	Lipids (prenol lipids-triterpenes)
Cholestadiene	25.55	C_27_H_44_	747-90-0	368.3437 – [C_27_H_44_]^+^ 247.2412 – [C_18_H_31_]^+^ 147.1141 – [C_11_H_15_]^+^	Lipids (steroids and derivatives)
Triethyl citrate	14.40	C_12_H_20_O_7_	77-93-0	203.0913 – [C_9_H_15_O_5_]^+^ 157.0496 – [C_7_H_9_O_4_]^+^ 83.0486 – [C_5_H_7_O]^+^	Organic acids and derivatives (carboxylic acids and derivatives)
Spiro [2,4]heptane-1,5-dimethyl-6-methylene	5.95	C_10_H_16_	62238-24-8	136.1225 – [C_10_H_16_]^+^ 121.0990 – [C_9_H_3_]^+^ 79.0524 – [C_6_H_7_]^+^	Other organic compounds
2-Propanol 1-(2-butoxy-1-methylethoxy)-	9.21	C_10_H_22_O_3_	29911-28-2	59.0485 – [C_3_H_7_O]^+^ 86.0715 – [C_5_H_10_O]^+^ 103.0728 – [C_5_H_11_O_2_]^+^	Other organic compounds
2,4,6-Triisopropylphenol	12.69	C_17_H_26_O	08-07-34	220.1822 – [C_15_H_24_O]^+^ 205.1584 – [C_14_H_21_O]^+^ 77.0369 – [C_6_H_5_]^+^	Other organic compounds
1,3-Heptadecyn-1-ol	15.11	C_17_H_32_O	56554-77-9	225.1826 – [C_14_H_25_O_2_]^+^ 81.0681 – [C_6_H_9_]^+^ 67.0529 – [C_5_H_7_]^+^	Other organic compounds
2,4-Diphenyl-4-methyl-2(E)-pentene	16.45	C_18_H_20_	22768-22-5	236.1567 – [C_18_H_20_]^+^ 143.0809 – [C_11_H_11_]^+^ 91.0513 – [C_7_H_7_]^+^	Other organic compounds
10,18-Bisnorabieta-8,11,13-triene	17.99	C_18_H_26_	32624-67-2	242.2007 – [C_18_H_26_]^+^ 227.1790 – [C_17_H_23_]^+^ 143.0864 – [C_11_H_11_O]^+^	Other organic compounds
2,6-Di-*t*-butyl-4-(2-phenylpropan-2-yl)phenol	18.71	C_23_H_32_O	34624-81-2	324.2438 – [C_23_H_32_O]^+^ 309.2212 – [C_22_H_29_O]^+^ 119.0836 – [C_9_H_11_]^+^	Other organic compounds
Phenol 2,2′-methylenebis[6-(1,1-dimethyl)-4-methyl	21.77	C_23_H_32_O_2_	119-47-1	330.1984 – [C_24_H_26_O]^+^ 315.1748 – [C_23_H_23_O]^+^ 237.1263 – [C_17_H_17_O]^+^	Other organic compounds
Phenol 2,4-bis(1-methyl-1-phenylethyl)-	22.52	C_24_H_26_O	2772-45-4	330.1984 – [C_24_H_26_O]^+^ 315.1748 – [C_23_H_23_O]^+^ 237.1263 – [C_17_H_17_O]^+^	Other organic compounds
2,4-Bis(dimethylbenzyl)-6-*t*-butylphenol	22.57	C_28_H_34_O	244080-16-8	386.2617 – [C_28_H_34_O]^+^ 371.2370 – [C_27_H_31_O]^+^ 293.1897 – [C_21_H_25_O]^+^	Other organic compounds
*n*-Hexadecylindane	23.93	C_25_H_42_	55334-29-7	117.0351 – [C_9_H_9_]^+^ 130.0427 – [C_10_H_10_]^+^ 154.1345 – [C_11_H_22_]^+^	Other organic compounds

The KEGG (Kyoto Encyclopedia of Genes and Genomes) database was used for identification of the main changes occurring in the composition of EBC from individuals pertaining to each class.

## Results

### Comparison of the EBC composition from current smokers and never smokers

**Figure 1:**
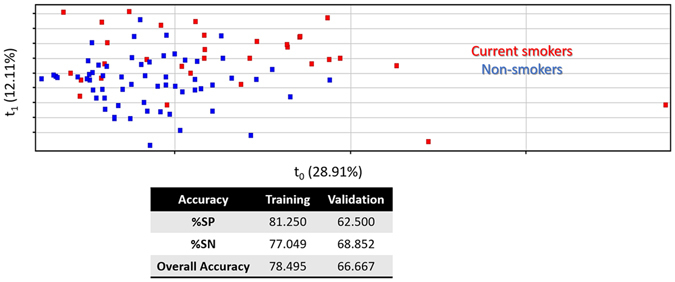
Supervised Analysis by PLS-DA built from the data set obtained after analysis of EBC extracts comparing current smokers and non-smokers.

As mentioned above, the present research is focused on detecting modifications in EBC composition associated to smoking habit. With this aim statistical analysis was carried out to compare the composition of EBC from CS and NS individuals. Supervised analysis by PLS-DA was applied to find differences in EBC collected from the two groups with the data set including the 44 identified compounds. This analysis would allow finding discrimination patterns associated to the smoking habit. Figure 1 illustrates the 3D scores plots, in which discrimination trends were clearly observed highlighting the influence of smoking habit on data variability. The percentage of correctly classified samples for the training set of the resulting PLS-DA were 81.3 and 77.1% for CS and NS individuals, respectively; while the validation set gave an overall accuracy of 66.7%. Therefore, there is a variability pattern in the EBC composition associated to the smoking habit. Statistical analysis by the *t*-test enabled to identify twelve compounds present at significantly different concentrations in CS and NS individuals with *p*-value below 0.05. Among these compounds, listed in Table 3, it is worth mentioning five that were highly significant, with a *p*-value below 0.01: indole (*p*-value 0.0006), undecanol (0.0036) and three phenolic compounds such as *p*-cresol (0.0065), 2,4,6-tris(1-methylethyl)-phenol (0.0032) and 2,6-bis(1,1-dimethylethyl)-4-(1-methyl-1-phenylethyl)-phenol (0.0031). Table 3 also includes the fold change ratio for these compounds by considering the two groups of individuals, CS and NS. It is worth mentioning that all the compounds were characterized by a similar concentration profile, with higher relative concentration in EBC from NS individuals as compared to CS, except for one compound, monostearin, that was more concentrated in CS. Special emphasis should be paid in terms of fold change ratio to indole, the most significant compound (*p*-value < 0.001), which, apart from its high significance, led to a fold change of 2.59, being more concentrated in NS individuals. Monostearin, a significant compound (0.05 < *p*-value < 0.01), presented the highest fold change value, 2.63, being more concentrated in the CS group. *p-*Cresol, also highly-significant (0.01 < *p*-value < 0.001), reported the third value in terms of fold change ratio (2.30) with a more concentrated level in EBC from NS individuals. The rest of significant compounds in the comparison between CS and NS groups provided fold change ratios from 1.67 to 1.10.

**Table 3:** Significant compounds from the statistical analysis by *t*-test comparing CS and NS individuals.

Compound (NS *vs* CS)	*p*-Value	Fold Change	Regulation
*p*- Cresol	0.0065	2.30	Up
Camphor	0.0156	1.25	Up
Indole	0.0006	2.59	Up
Undecanol	0.0036	1.51	Up
2,4,6-Tris(1-methylethyl)-phenol	0.0032	1.21	Up
Benzoic acid 4-ethoxy-ethyl ester	0.0410	1.10	Up
2,4-Diphenyl-4-methyl-2(E)-pentene	0.0416	1.30	Up
2,6-Bis(1,1-dimethylethyl)-4-(1-methyl-1-phenylethyl)-phenol	0.0031	1.67	Up
Stearic acid	0.0152	1.52	Up
2,4-Bis(1-methyl-1-phenylethyl)-phenol	0.0127	1.26	Up
2,4-Bis(dimethylbenzyl)-6-*t*-butylphenol	0.0119	1.19	Up
Monostearin	0.0350	2.63	Down

**Figure 2:**
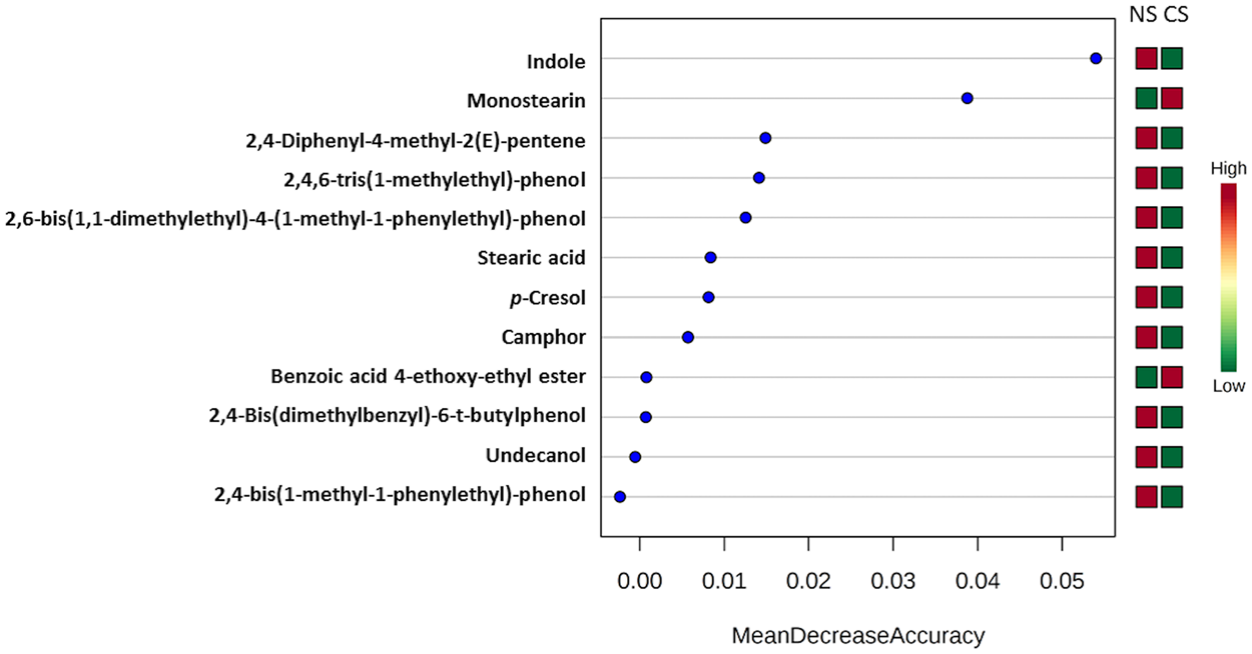
Random Forest Analysis built from the data set obtained after analysis of EBC from CS and NS groups.

**Figure 3:**
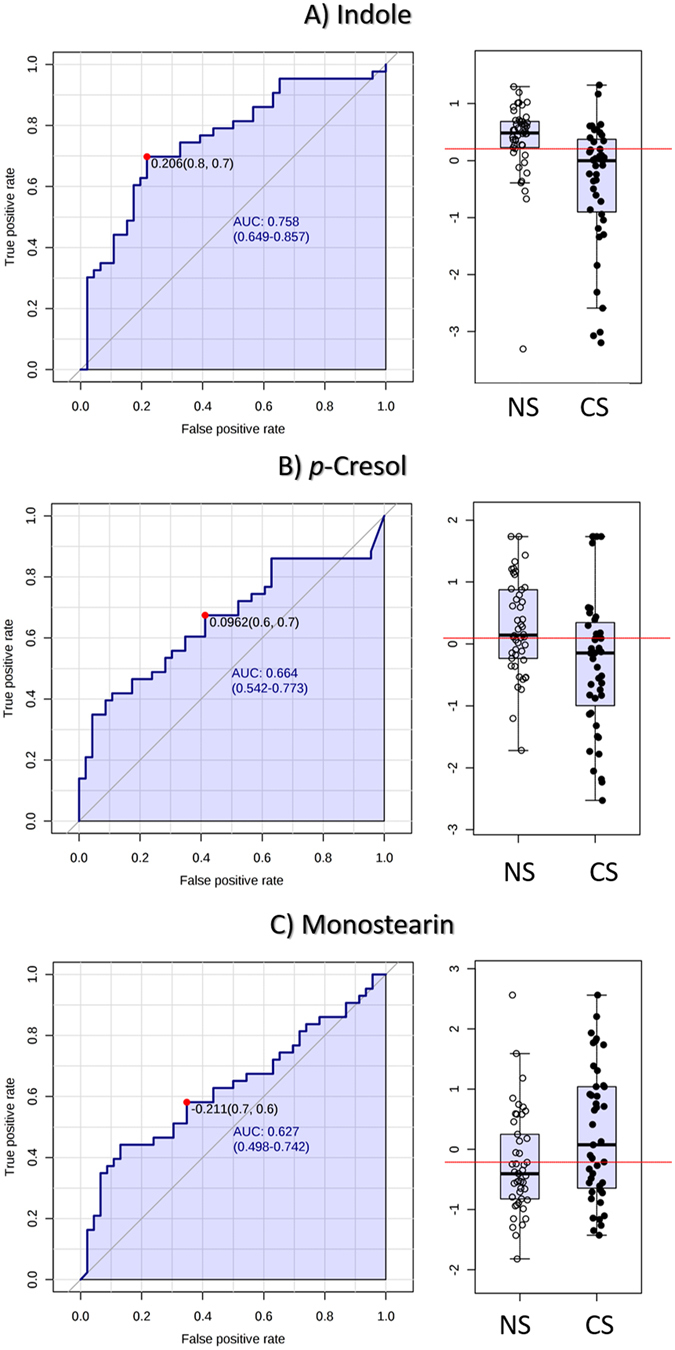
ROC curves and Box and Whiskers plots for the three compounds with the highest significance, fold change and capability for discrimination in the comparison between CS and NS groups.

Random Forest Analysis was applied to compare the discrimination accuracy of significant compounds in this study dealing with CS and NS groups. The ranking of compounds according to discrimination accuracy is shown in Fig. 2 that enables to identify indole and monostearin as the two compounds with the highest discrimination capability as compared to the rest. The discrimination accuracy of these compounds was also checked by analysis of ROC curves, as shows Fig. 3. *p*-Cresol was also included in this analysis due to its high significance and fold change ratio. As can be seen, indole reported the best predictive behavior, with a value of area under the curve (AUC) of 0.758, and specificity/sensitivity values of 0.8/0.7. This good performance can be checked in the box-and-whisker plot with the cut-off value for discrimination between CS and NS groups. Monostearin and *p*-cresol reported similar AUC values, 0.664 and 0.627, respectively, and close specificity/sensitivity parameters, 0.7/0.6 and 0.6/0.7, respectively. With these premises, it is worth emphasizing the statistical contribution of these three compounds to explain the differences in composition of EBC from CS and NS individuals.

### Comparison of the composition of EBC from current smokers and former smokers

**Figure 4:**
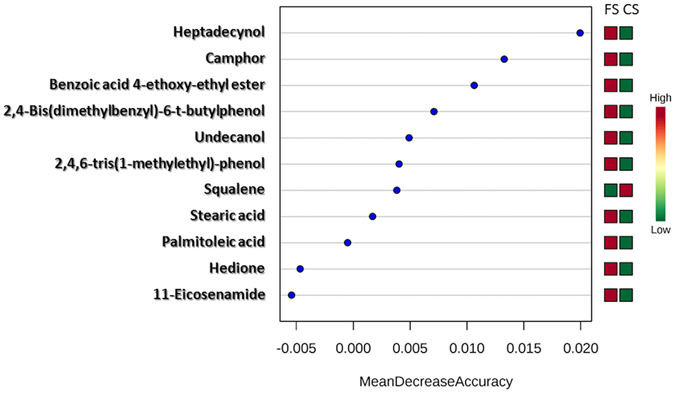
Ranking of predictive capability of compounds to discriminate between CS and FS individuals.

**Figure 5:**
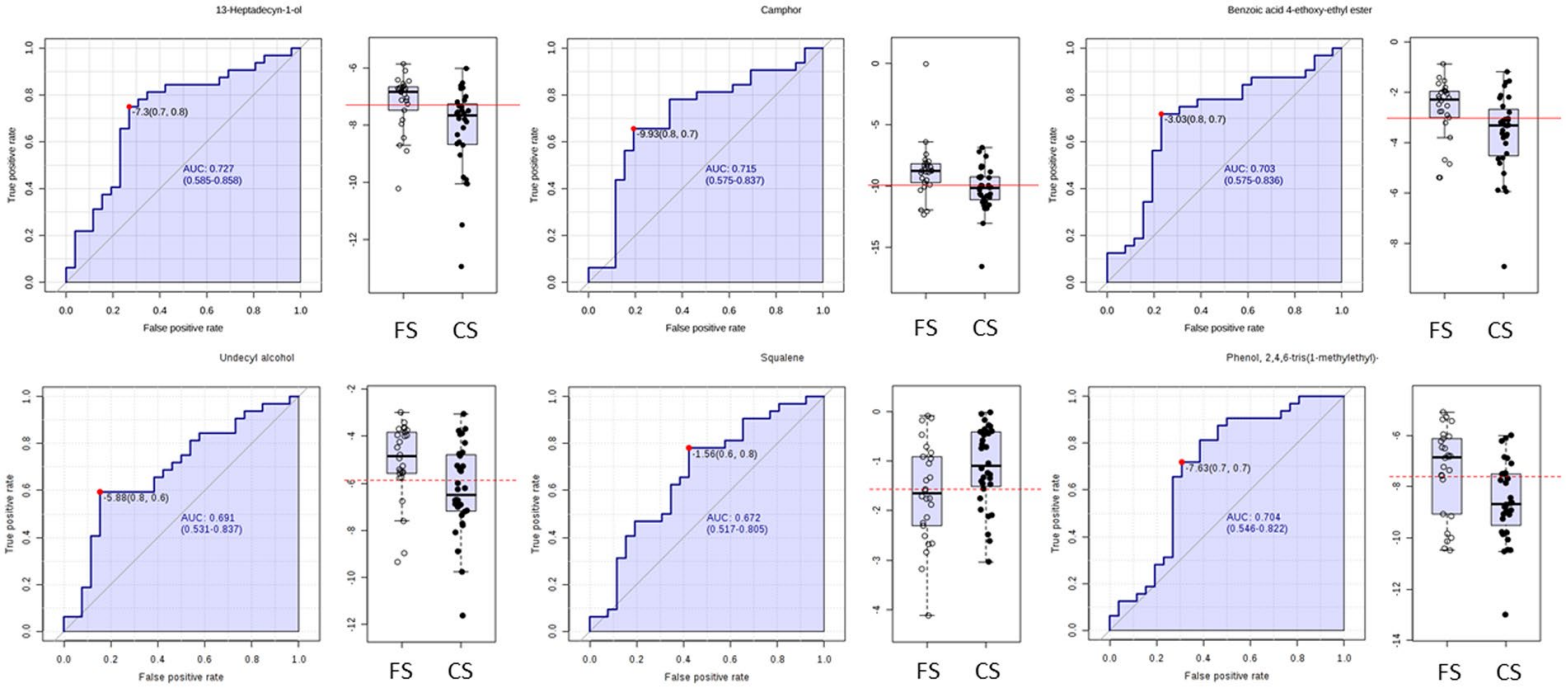
ROC curves and box-and-whiskers plot for the six compounds with the highest significance and predictive capability to discriminate CS from FS individuals.

In this step, the composition of EBC from CS individuals was compared to that from FS individuals to evaluate the incidence of quitting smoking on EBC composition. Two relevant factors such as the smoking habit and the cumulative consumption, expressed as smoked pack-year, were considered as categorical variables in this study. Concerning the smoking habit, the individuals were divided into two groups —namely, FS (>1 year they quit smoking) and CS. This classification allowed obtaining balanced groups. Three equilibrated groups were also considered to study the effect of the cumulative consumption as smoked pack-year (0–24, 24–45, >45 packs-year). Supplementary Table 1 shows the number of individuals classified in each group. A two-factor ANOVA was applied to detect EBC compositional changes according to the two parameters. The analysis of the smoking habit reported eleven significant compounds with *p*-values below 0.05, which are listed in Table 4. Among the compounds, it is worth mentioning the presence of six highly-significant (*p*-value < 0.01) compounds such as camphor (*p*-value 0.007), undecanol (0.0100), heptadecynol (0.0054), squalene (0.0080) and two phenolic derivatives: 2,4,6-tris(1-methylethyl)-phenol (0.0056) and benzoic acid 4-ethoxy-ethyl ester (0.0048). In all cases, the levels of these compounds were higher in EBC from FS than from CS, except for squalene that reported the opposite situation being at higher level in EBC from CS. Figure 4 shows the ranking of predictive capability of compounds to discriminate between CS and FS groups. As can be seen, the compounds that reported the highest statistical significance (*p*-value < 0.01) were in the top-7 compounds according to the ranking provided by the Random Forest Analysis. The discrimination accuracy of these compounds can be visualized in Fig. 5 that shows the ROC curve for each compound. Thus, heptadecynol and camphor provided AUC values of 0.727 and 0.715, respectively; while specificity/sensitivity were 0.7/0.8 and 0.8/0.7, respectively. The two phenol derivatives benzoic acid 4-ethoxy-ethyl ester and 2,4,6-tris(1-methylethyl)-phenol gave a similar AUC value, while the specificity/sensitivity values were 0.8/0.7 and 0.7/0.7, respectively. Finally, undecanol and squalene reported AUC values slightly below 0.7, while the specificity/sensitivity values were 0.8/0.6 and 0.6/0.8, respectively.

**Table 4:** Significant compounds from the statistical analysis by *t*-test comparing CS and FS individuals.

Compounds (CS vs FS)	*p*-value	Fold Change	Regulation
Camphor	0.0070	2.75	Down
Undecanol	0.0100	2.24	Down
2,4,6-tris(1-methylethyl)-phenol	0.0056	2.23	Down
Benzoic acid 4-ethoxy ethyl ester	0.0048	1.92	Down
Hedione	0.0419	1.81	Down
13-Heptadecynol	0.0054	1.94	Down
Palmitoleic acid	0.0166	1.80	Down
Stearic acid	0.0234	1.46	Down
2,4-Bis(dimethylbenzyl)-6-t-butylphenol	0.0270	1.50	Down
Eicosenamide	0.0372	1.22	Down
Squalene	0.0080	1.46	Up

Concerning the cumulative consumption, only five compounds were significant by comparing their EBC levels in the three groups: three terpenoids —limonene (0.0124), eucalyptol (0.0071) and levomenthol (0.0209)— together with benzyl alcohol (0.0096) and spiro[2,4]heptane,1,5-dimethyl (0.0114). According to these parameters, only eucalyptol and benzyl alcohol reported statistical differences with 99.9% of confidence. The five significant compounds were characterized by the same pattern, except for levomenthol. Thus, the EBC levels of these compounds were higher in smokers with an intermediate consumption, except for levomenthol that showed similar levels in smokers with intermediate and high cumulative consumption.

## Discussion

### Comparison of the composition of EBC from current smokers and never smokers

In addition to be the most relevant risk factor in respiratory diseases and several types of cancer, smoking is also a key risk factor in several diseases such as cardiovascular diseases. As previously emphasized, numerous biochemical changes occurring in CS as compared to NS individuals have been described, including those supported on metabolomics analysis. However, most of these studies have been focused on conventional biofluids such as serum/plasma, and few of them have been carried out with less conventional samples such as sputum or exhaled breath. The present research was targeted at the analysis of EBC that is obtained in a non-invasive manner and can be easily handled due to condensation in a liquid phase. The first preliminary attempt was to find discrimination patterns in EBC sampled from CS and NS in the controlled cohort of volunteers enrolled at the Department of Respiratory Medicine of the Reina Sofía Hospital. A PLS-DA analysis allowed finding a discrimination pattern between both groups of individuals. In fact, the PLS accuracy parameters of the resulting model were consistent for the training and validation steps. This result confirmed the existence of differences in the composition of EBC from CS and NS. The differences could be associated to three main changes: (1) alterations of endogenous metabolism —for example, increase of inflammatory markers owing to free radicals formation; (2) exogenous compounds owing to the smoking habit such as those described in the extensive research carried out by Filipiak *et al*.^25^; (3) compounds associated to microbiome-host interactions, which could also be linked to the first group.

Most of the microbiome research carried out to date has focused on bacterial communities in the colon, the site of the largest, most complex and active microbial system in the body. However, polymicrobial communities exist in many other body sites, including the skin^26^, the mouth^27, 28^, genitourinary^29^ and respiratory tracts^30, 31^. In each case, bidirectional communication between microbes and the underlying tissue regulates the local environment, including physical characteristics as well as the immune response. Microbiome dysbiosis is strongly associated with certain pathologies although the determinants of these microbial imbalances, particularly in body sites different from colon, are largely unknown.

Targeting at the respiratory tract the disruption of microbial communities has been implicated in dysregulation of local immunity^32^, infection susceptibility^31^, and the development of chronic respiratory inflammatory diseases^33^. As an example, specific bacterial species are implicated in the pathogenesis of exacerbations of chronic obstructive pulmonary disease (COPD), which seems to be associated with accelerated loss of lung function in COPD. Recent studies of clinically stable COPD patients have demonstrated a greater diversity of airway microbiota as compared to healthy individuals. Also, Huang *et al*. observed the sputum bacterial composition to be generally stable over the preexacerbation period of clinical stability, but to change at the time of exacerbation, with specific enrichment in not only typical COPD-associated bacterial species (*e.g*., Haemophilus influenzae) but also in other phylogenetically related species with pathogenic potential such as Gammaproteobacteria, Delta- and Betaproteobacteria^34^. Concurrently, these authors found depleted abundance of other bacteria such as Firmicutes and Actinobacteria phyla whose predicted metagenomes suggest functional capacities to produce a variety of anti-inflammatory compounds such as betalain, flavonoids, macrolides and indole alkaloids. This result is in agreement with EBC levels found in this research for indole, the compound that reported the highest significance (*p*-value 0.0006) comparing CS and NS with a fold change of 2.59, being more concentrated in NS individuals. Therefore, smoking habit leads to the modification of the respiratory microbiome with incidence on the immune response, which is here supported at metabolomics level on EBC concentrations of indole, a metabolite with recognized anti-inflammatory and antimicrobial properties. The alteration of the immune response contributes to explain the susceptibility of CS to respiratory diseases. This biological interpretation gains interest by the predictive behavior of indole to discriminate CS from NS individuals. As can be seen, the ROC curve for this metabolite reported a high AUC value with values of specificity and sensitivity relatively high: 80 and 70%, respectively.

A representative group of compounds was phenols and derivatives, which also were characterized by a high significance in the comparison of their levels in EBC from CS and NS individuals. The most relevant compound was *p*-cresol (0.0065), involved in the degradation of toluene. Toluene is mainly metabolized to benzyl alcohol or to hydroxytoluene isomers, particularly to *p-*cresol. This compound, also endowed with antioxidant properties by virtue of the phenolic functional group, was also found at higher levels in EBC from NS individuals. This result strongly agrees with that found by Wu *et al*.^35^, who found different oral microbiome composition between current and non-current (former and non) smokers (*p* < 0.001). CS individuals presented lower relative abundance of the phylum Proteobacteria (4.6%) as compared with NS individuals (11.7%). Taxa not belonging to Proteobacteria were also associated with smoking. Thus, the genera Capnocytophaga, Peptostreptococcus and Leptotrichia were depleted, while Atopobium and Streptococcus were enriched in CS compared with NS individuals. Functional analysis from inferred metagenomes showed that bacterial genera depleted by smoking were related to carbohydrate and energy metabolism, and to xenobiotic metabolism such as that of toluene. Particularly, toluene metabolism was more active in the genera of bacteria depleted in CS than in bacteria that are enriched in this group of individuals.

A similar pattern was found for the two alkylphenol derivatives, 2,4,6-tris(1-methylethyl)-phenol (0.0032) and 2,6-bis(1,1-dimethylethyl)-4-(1-methyl-1-phenylethyl)-phenol (0.0031). These two compounds are structurally similar to a known antioxidant widely used in the food and cosmetic industries: butylated hydroxytoluene (BTH). Both compounds could be formed through the degradation of exogenous compounds by the microbiome, which could explain the different levels in EBC from CS and NS. The structural analogy between the two alkylphenols and BTH allows deducing an antioxidant activity of these two compounds. There was one other highly significant compound in EBC between CS and NS: undecanol (0.0036), which has not previously been related to the microbiome. Some groups have described the potential formation of fatty alcohols through degradation of polyunsaturated fatty acid (PUFA) metabolites that are considered inflammation markers^36^. Some of these compounds are endowed with anti- or pro-inflammatory properties depending on the PUFA precursor. In this specific case, undecanol was present at higher level in EBC from NS individuals than from CS, which potentially could explain its formation through PUFA metabolites with anti-inflammatory capability.

Special emphasis should be also put on monostearin, which reported less significance (0.05 < *p*-value < 0.01) between CS and NS, but experienced the highest fold change value, 2.63, at higher levels in EBC from CS. There are not evidences about relationships of monostearin levels in individuals affected by respiratory diseases or smoking habit as compared to healthy individuals or NS, respectively. Wikoff *et al*. found significantly lower level of monostearin in malignant lung tissue from early stage adenocarcinoma patients as compared to non-malignant tissue, while monopalmitin reported the opposite situation^37^. Levels in lung tissue and EBC could be correlated due to the absorption/desorption equilibrium during breathing.

### Comparison of EBC composition from current smokers and former smokers

A recent research suggested that the oral microbiome appears to return to its previous state for FS individuals. The microbial balance in individuals who quit smoking for more than 10 years was similar to that observed in NS individuals^1^. However, the results did not reveal the time required to recover the microbiome balance. In the research proposed here an additional aim was to find discrimination patterns in EBC sampled from CS and FS in the controlled cohort of volunteers enrolled at the Department of Respiratory Medicine of the Reina Sofía Hospital. For this purpose, the smoking load was also taken into account as a categorical factor. Concerning the smoking habit 6 compounds resulted highly significant in the comparison between CS and FS individuals: camphor, undecanol, heptadecynol, squalene and two phenol derivatives —2,4,6-tris(1-methylethyl)-phenol and benzoic acid 4-ethoxy-ethyl ester. Squalene is a naturally occurring polyprenyl compound known for its key role as an intermediate in cholesterol synthesis. Many other polyprenyl compounds structurally similar to squalene are detected in biofluids and perform critical biological functions such as oxidation inhibition or antimicrobial properties. For example, animals utilize prenyl groups to form the side chain of ubiquinones, among which it is worth emphasizing the role of coenzyme Q10 (the most common form of ubiquinone in the human body) as endogenous antioxidant. Other well-known prenyl derivatives include carotenes, vitamin A, vitamin K, vitamin E and cyclic terpenoid compounds such as camphor, pinene and limonene^38^. Thus, the significant different concentration of camphor and squalene in CS and FS individuals (squalene is more concentrated in CS than in FS and camphor shows the opposite trend) could be attributed to the fact that quitting smoking stimulates the synthesis of compounds with antimicrobial properties like camphor using prenyl groups from squalene. In addition, as discussed above, smoking habit upsets the immune response contributing to explain the susceptibility of CS to respiratory diseases. This biological interpretation is supported on the predictive behavior of both compounds to discriminate between CS and FS. As can be seen, the ROC curves for these metabolites reported a high AUC value with relatively high specificity and sensitivity, 80 and 70% for camphor and 60 and 80% for squalene, respectively. These results could be related to that found by Biedermann *et al*., who investigated the role of smoking cessation on intestinal microbial composition in 10 healthy smokers undergoing controlled smoking cessation^39^. They found an increase of sequences from *Firmicutes* and *Actinobacteria* and a simultaneous decrease of the *Proteobacteria* and *Bacteroidetes* fractions after smoking cessation. This behavior was related to the production of a variety of anti-inflammatory and antimicrobial compounds.

Two other highly significant compounds in EBC from CS and FS individuals were undecanol (0.0100) and heptadecynol (0.0054) that have not been previously related to the microbiome. As discussed above, the formation of these fatty alcohols could be related to PUFA metabolites degradation. Both compounds were present at higher level in EBC from FS than from CS individuals, which potentially could explain their source through PUFA metabolites with anti-inflammatory capability.

One other significant compound was an alkylphenol derivative, 2,4,6-tris(1-methylethyl)-phenol (0.0056) which was discussed in the previous section as a BTH related compound with antioxidant activity. This compound presented higher levels in FS than in CS individuals, supporting the results obtained comparing CS with NS individuals. Special emphasis should be also put on benzoic acid 4-ethoxy ethyl ester, which reported the highest significance between CS and FS individuals (0.0048). This compound had previously been found in exhaled breath^40^, but there are not evidences about its relationship with the smoking habit. The depletion of certain xenobiotic biodegradation pathways in CS suggests important functional losses with potential health consequences^1^. Oral bacteria are first to come into contact with cigarette smoke and may play an important role in degrading the accompanying toxic compounds. Wu *et al*. observed that functional pathways related to toluene, nitrotoluene, styrene, chlorocyclohexane and chlorobenzene degradation were depleted in CS as well as the cytochrome P450 xenobiotic metabolism^1^. Conversely, polycyclic aromatic hydrocarbon and xylene degradation were enriched in CS individuals. However, Meckenstock *et al*. concluded in their research that toluene was the responsible for the metabolic inhibition of xylene-degrading organisms^41^. This result could be in agreement with the high level of benzoic acid 4-ethoxy ethyl ester found in EBC from FS individuals. Quitting smoking reduces the presence of toluene levels stimulating xylene degradation and increasing the level of benzoic acid derivatives.

Concerning the smoking cumulative consumption, benzyl alcohol resulted highly significant (0.0096) as well as eucalyptol (0.0071). Benzyl alcohol is one of the two intermediates in the pathway of toluene metabolism with a trend opposite to *p-*cresol. Therefore, the benzyl alcohol levels increased with smoking load, which agrees with *p-*cresol EBC levels that were higher for NS than for CS individuals.

Eucalyptol is the bicyclic monoterpene 1,8-cineole, which is transformed by several pathways. *Novosphingobium subterranea* converts 1,8-cineole initially into 2-endo-hydroxycineole, 2,2-oxo-cineole and 2-exo-hydroxycineole. A cytochrome P450 monooxygenase catalyzes the hydroxylation of 1,8-cineole; thus, the higher levels of eucalyptol with the increase of the smoking load is explained by the cytochrome P450 xenobiotic metabolism, depleted in CS individuals, favoring the accumulation of eucalyptol as a result.

### Limitations of the study

The following two aspects should be considered to interpret the obtained results:

1. Spirometry was not performed in the target cohort and, thus, no data about lung function were used for correlation with significant compounds. Decline in lung function in smokers and its relationship with EBC significant compounds cannot be ruled out. Nevertheless, the range of collected volume was quite narrow (1.25–1.75 mL) and, additionally, relative concentrations were used by MSTUS approach to remove the effect of the sampled volume.
2. BMI was higher than 30 kg/m^2^ in 42% of individuals (Supplementary Table 1) who have included in respective groups only for categorization of subjects and their physiological data. These individuals were distributed into the three groups considered in this research without statistical significance. Hence, the obtained results should be cautiously interpreted to associate the smoking habit with metabolite composition.

## Conclusions

Smoking habit is one of the risk factors associated to multiple respiratory diseases and it may influence EBC composition. In this research, 12 metabolites have been found at significantly different levels between current smokers and non-smokers. Among them it is worth mentioning monostearin, indole and *p*-cresol due to their high significance. Additionally, 12 metabolites have been found significantly different between current and former smokers, including compounds with structure similar to the previous case. In fact, 6 compounds were significantly different in both studies. Despite the necessity of a large-scale study to validate the EBC applicability to detect metabolic changes caused by smoking habit, this first approach has revealed statistically significant differences associated to the smoking habit.

## Electronic supplementary material

Supplementary info
