# Supplementary material for: Exhaled breath condensate to discriminate individuals with different smoking habits by GC–TOF/MS
Source: Sci Rep. 2017 May 3;7:1421. doi: 10.1038/s41598-017-01564-z (PMC5431160; doi:10.1038/s41598-017-01564-z)
Supplement: Supplementary file 1 — Supplementary info [file 41598_2017_1564_MOESM1_ESM.doc]

**Exhaled breath condensate to discriminate individuals with different smoking habits by GC–TOF/MS**

A. Peralbo-Molina^a,b^, M. Calderón-Santiago^a,b^, B. Jurado-Gámez^b,c^, M.D. Luque de Castro^a,b*^, F. Priego-Capote^a,b*^

**
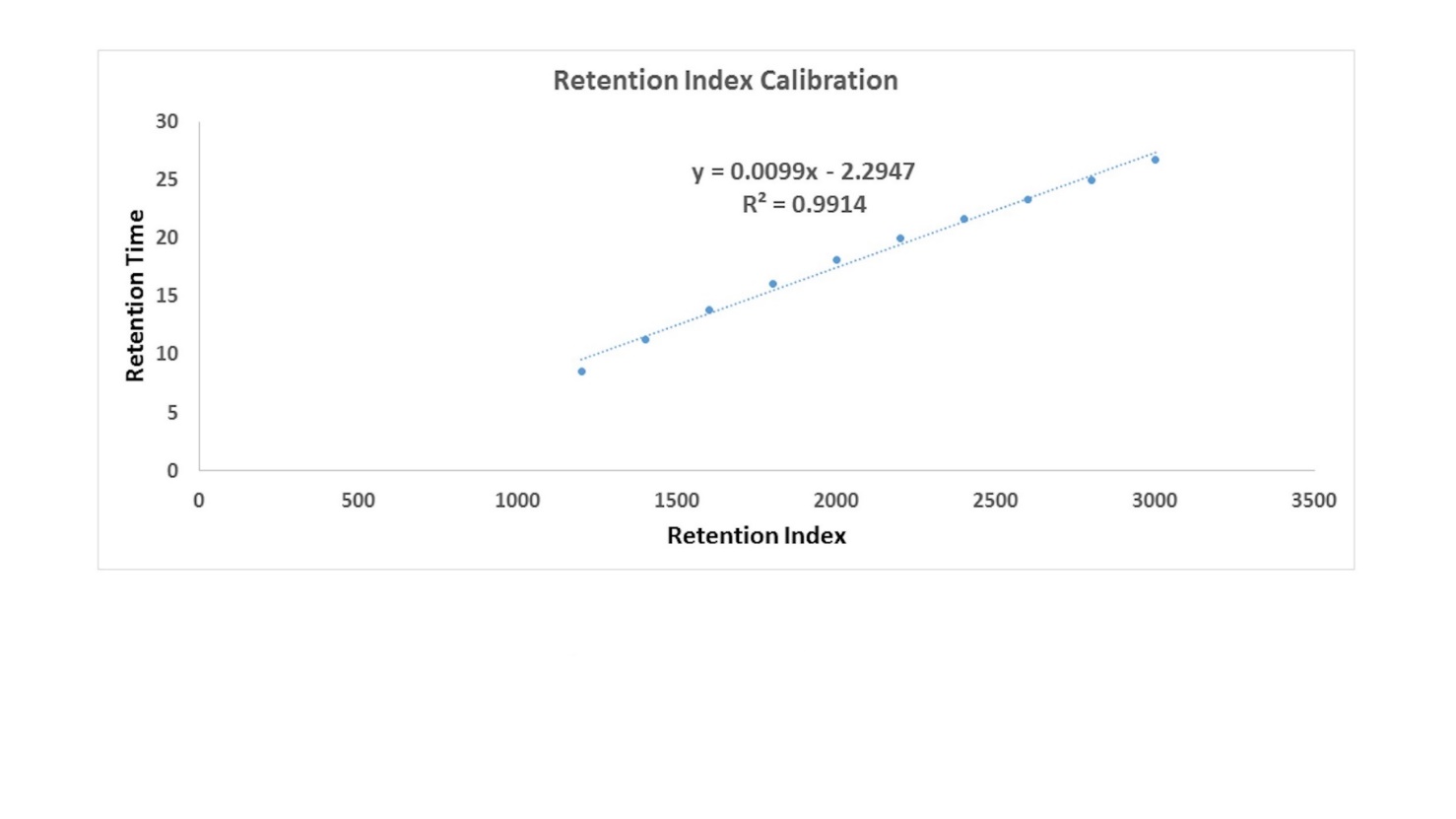
**

**Supplementary Figure 1.** RI calibration curve from analysis of the alkane standard mixture.

**Supplementary Table 1.** Data from each individual such as age, sex and cigarettes consumption expressed as smoked pack-year.

| **CODE** | **Gender** | **Age** | **Weight** | **Heigth** | **BMI** | **Smoking Habits** | **Smoking Load** |
| --- | --- | --- | --- | --- | --- | --- | --- |
| **OV14-01-052** | Female | 51 | 52 | 1.65 | 19.1 | CS | 25-44 |
| **OV14-04-002** | Male | 57 | 61.5 | 1.73 | 20.55 | NS | 0 |
| **OV13-04-071** | Female | 54 | 57.7 | 1.66 | 20.94 | NS | 0 |
| **OV14-01-106** | Female | 51 | 57 | 1.62 | 21.72 | NS | 0 |
| **OV13-01-020** | Male | 67 | 63 | 1.7 | 21.8 | CS | >45 |
| **OV13-04-202** | Male | 48 | 74.5 | 1.81 | 22.74 | NS | 0 |
| **OV14-01-082** | Male | 51 | 60 | 1.62 | 22.86 | CS | >45 |
| **OV14-01-049** | Male | 59 | 69 | 1.72 | 23.32 | FS | >45 |
| **OV13-04-114** | Male | 44 | 71.5 | 1.74 | 23.62 | CS | 25-44 |
| **OV13-01-100** | Male | 57 | 76 | 1.79 | 23.72 | NS | 0 |
| **OV13-04-138** | Male | 52 | 77 | 1.8 | 23.77 | CS | 25-44 |
| **OV13-01-119** | Male | 70 | 73 | 1.75 | 23.84 | FS | 25-44 |
| **OV14-01-071** | Male | 60 | 65 | 1.65 | 23.88 | NS | 0 |
| **OV14-01-117** | Male | 61 | 77 | 1.79 | 24.03 | NS | 0 |
| **OV14-01-070** | Male | 51 | 78 | 1.8 | 24.07 | NS | 0 |
| **OV14-01-075** | Female | 66 | 55 | 1.5 | 24.44 | CS | 25-44 |
| **OV14-01-105** | Male | 52 | 75 | 1.75 | 24.49 | CS | 0-24 |
| **OV14-01-079** | Male | 54 | 82 | 1.83 | 24.49 | FS | 25-44 |
| **OV13-04-215** | Male | 58 | 67.5 | 1.66 | 24.5 | NS | 0 |
| **OV14-01-094** | Male | 56 | 70 | 1.69 | 24.51 | NS | 0 |
| **OV13-04-166** | Male | 50 | 71 | 1.7 | 24.57 | NS | 0 |
| **OV13-04-060** | Male | 51 | 77 | 1.77 | 24.58 | FS | >45 |
| **OV14-01-058** | Female | 57 | 67 | 1.65 | 24.61 | NS | 0 |
| **OV13-04-214** | Male | 51 | 72 | 1.71 | 24.62 | NS | 0 |
| **OV13-04-120** | Male | 56 | 76.5 | 1.76 | 24.7 | CS | 0-24 |
| **OV13-04-088** | Male | 63 | 66.5 | 1.64 | 24.72 | FS | 0-24 |
| **OV14-01-026** | Male | 69 | 70 | 1.68 | 24.8 | CS | 25-44 |
| **OV13-01-130** | Male | 56 | 61 | 1.56 | 25.07 | CS | 0-24 |
| **OV13-04-113** | Male | 43 | 73.5 | 1.7 | 25.43 | NS | 0 |
| **OV13-01-074** | Male | 60 | 71 | 1.67 | 25.46 | CS | >45 |
| **OV14-01-033** | Female | 71 | 66 | 1.6 | 25.78 | NS | 0 |
| **OV13-04-008** | Female | 60 | 68 | 1.62 | 25.91 | FS | 0-24 |
| **OV13-04-076** | Male | 55 | 78.4 | 1.73 | 26.2 | NS | 0 |
| **OV14-01-044** | Male | 54 | 85 | 1.8 | 26.23 | FS | 25-44 |
| **OV13-01-099** | Male | 55 | 76 | 1.7 | 26.3 | NS | 0 |
| **OV13-01-110** | Male | 66 | 76 | 1.7 | 26.3 | CS | 0-24 |
| **OV13-04-058** | Female | 55 | 70 | 1.63 | 26.35 | NS | 0 |
| **OV13-04-077** | Male | 65 | 81.7 | 1.76 | 26.38 | FS | 0-24 |
| **OV13-04-004** | Female | 65 | 68 | 1.6 | 26.56 | NS | 0 |
| **OV13-04-075** | Female | 70 | 69 | 1.61 | 26.62 | NS | 0 |
| **OV13-01-116** | Male | 64 | 82 | 1.75 | 26.78 | CS | >45 |
| **OV13-04-132** | Male | 46 | 79.9 | 1.72 | 27.01 | CS | 0-24 |
| **OV13-04-081** | Male | 53 | 80 | 1.72 | 27.04 | NS | 0 |
| **OV13-04-103** | Male | 67 | 65 | 1.55 | 27.06 | NS | 0 |
| **OV13-01-095** | Male | 56 | 85 | 1.77 | 27.13 | CS | 25-44 |
| **OV14-01-076** | Male | 61 | 84 | 1.75 | 27.43 | NS | 0 |
| **OV14-01-091** | Male | 60 | 73 | 1.63 | 27.48 | NS | 0 |
| **OV14-01-034** | Male | 72 | 75 | 1.65 | 27.55 | NS | 0 |
| **OV14-01-029** | Female | 63 | 62 | 1.5 | 27.56 | FS | 0-24 |
| **OV14-01-077** | Male | 68 | 80 | 1.7 | 27.68 | FS | 25-44 |
| **OV13-04-123** | Male | 45 | 79.5 | 1.69 | 27.84 | NS | 0 |
| **OV13-04-186** | Male | 74 | 70 | 1.58 | 28.04 | NS | 0 |
| **OV14-01-100** | Male | 62 | 83 | 1.72 | 28.06 | CS | 0-24 |
| **OV13-01-014** | Female | 72 | 63 | 1.49 | 28.38 | NS | 0 |
| **OV13-04-154** | Male | 56 | 87.5 | 1.75 | 28.57 | NS | 0 |
| **OV14-01-087** | Male | 58 | 78 | 1.65 | 28.65 | NS | 0 |
| **OV14-01-080** | Male | 54 | 78 | 1.65 | 28.65 | CS | 0-24 |
| **OV14-01-098** | Male | 51 | 88 | 1.75 | 28.73 | FS | 0-24 |
| **OV14-01-001** | Male | 75 | 88 | 1.75 | 28.73 | FS | >45 |
| **OV13-04-003** | Male | 56 | 69.3 | 1.55 | 28.84 | NS | 0 |
| **OV13-01-030** | Female | 53 | 75 | 1.61 | 28.93 | CS | 25-44 |
| **OV13-04-054** | Female | 47 | 77 | 1.63 | 28.98 | NS | 0 |
| **OV13-04-227** | Male | 71 | 88 | 1.74 | 29.07 | NS | 0 |
| **OV13-04-087** | Male | 66 | 85 | 1.71 | 29.07 | CS | 25-44 |
| **OV13-01-036** | Male | 57 | 105 | 1.9 | 29.09 | CS | >45 |
| **OV13-04-160** | Male | 56 | 89 | 1.74 | 29.4 | NS | 0 |
| **OV13-01-129** | Male | 55 | 75 | 1.59 | 29.67 | NS | 0 |
| **OV14-01-084** | Male | 59 | 94 | 1.78 | 29.67 | NS | 0 |
| **OV13-01-091** | Female | 63 | 78 | 1.62 | 29.72 | CS | 25-44 |
| **OV13-04-129** | Male | 52 | 83 | 1.66 | 30.12 | FS | 25-44 |
| **OV14-04-017** | Male | 57 | 83.6 | 1.66 | 30.34 | NS | 0 |
| **OV14-01-028** | Male | 59 | 91 | 1.72 | 30.76 | NS | 0 |
| **OV14-01-115** | Male | 50 | 102 | 1.82 | 30.79 | NS | 0 |
| **OV13-04-106** | Male | 53 | 94.5 | 1.75 | 30.86 | NS | 0 |
| **OV14-01-086** | Male | 59 | 93 | 1.73 | 31.07 | NS | 0 |
| **OV13-04-108** | Male | 44 | 87 | 1.67 | 31.2 | FS | 0-24 |
| **OV13-04-007** | Female | 59 | 81 | 1.61 | 31.25 | NS | 0 |
| **OV13-04-082** | Male | 58 | 101.5 | 1.8 | 31.33 | CS | 25-44 |
| **OV13-04-016** | Female | 52 | 49 | 1.25 | 31.36 | CS | 0-24 |
| **OV14-01-103** | Male | 62 | 93 | 1.72 | 31.44 | CS | >45 |
| **OV13-04-194** | Male | 53 | 100 | 1.78 | 31.56 | NS | 0 |
| **OV13-04-099** | Male | 53 | 102.3 | 1.8 | 31.57 | NS | 0 |
| **OV13-04-216** | Male | 47 | 87.5 | 1.66 | 31.75 | NS | 0 |
| **OV13-04-070** | Male | 61 | 86.5 | 1.65 | 31.77 | NS | 0 |
| **OV14-01-090** | Male | 51 | 94 | 1.72 | 31.77 | CS | 25-44 |
| **OV13-01-024** | Male | 59 | 96 | 1.73 | 32.08 | NS | 0 |
| **OV13-04-199** | Male | 77 | 89.5 | 1.67 | 32.09 | NS | 0 |
| **OV14-01-064** | Female | 58 | 80 | 1.57 | 32.46 | CS | 25-44 |
| **OV13-01-154** | Male | 64 | 114 | 1.87 | 32.6 | FS | 25-44 |
| **OV14-01-036** | Male | 56 | 100 | 1.75 | 32.65 | FS | 25-44 |
| **OV13-04-122** | Male | 74 | 88 | 1.64 | 32.72 | NS | 0 |
| **OV13-01-113** | Male | 75 | 87 | 1.63 | 32.74 | CS | >45 |
| **OV13-01-019** | Female | 59 | 85 | 1.61 | 32.79 | NS | 0 |
| **OV13-04-161** | Male | 65 | 80 | 1.56 | 32.87 | NS | 0 |
| **OV14-01-030** | Male | 68 | 74 | 1.5 | 32.89 | NS | 0 |
| **OV14-01-041** | Male | 60 | 94 | 1.69 | 32.91 | NS | 0 |
| **OV14-01-099** | Male | 57 | 100 | 1.74 | 33.03 | NS | 0 |
| **OV14-01-122** | Male | 65 | 82 | 1.57 | 33.27 | CS | >45 |
| **OV14-01-032** | Female | 68 | 78 | 1.53 | 33.32 | NS | 0 |
| **OV13-04-084** | Male | 62 | 93 | 1.67 | 33.35 | FS | 0-24 |
| **OV13-04-126** | Male | 72 | 92 | 1.66 | 33.39 | FS | 0-24 |
| **OV13-04-064** | Male | 61 | 99 | 1.72 | 33.46 | FS | >45 |
| **OV13-04-101** | Male | 54 | 97 | 1.7 | 33.56 | FS | >45 |
| **OV14-01-119** | Male | 73 | 95 | 1.68 | 33.66 | FS | >45 |
| **OV14-01-078** | Male | 52 | 100 | 1.72 | 33.8 | FS | >45 |
| **OV14-01-024** | Male | 69 | 100 | 1.72 | 33.8 | FS | >45 |
| **OV14-04-001** | Male | 72 | 104 | 1.75 | 33.96 | NS | 0 |
| **OV13-01-120** | Female | 49 | 94 | 1.66 | 34.11 | CS | 0-24 |
| **OV13-04-095** | Male | 59 | 112.5 | 1.81 | 34.34 | FS | 0-24 |
| **OV14-04-003** | Male | 63 | 100 | 1.7 | 34.6 | NS | 0 |
| **OV13-04-131** | Male | 49 | 100 | 1.7 | 34.6 | FS | 0-24 |
| **OV13-04-100** | Female | 63 | 88.5 | 1.57 | 35.9 | NS | 0 |
| **OV13-04-093** | Male | 58 | 102.5 | 1.68 | 36.32 | CS | 0-24 |
| **OV13-04-065** | Female | 67 | 91 | 1.58 | 36.45 | NS | 0 |
| **OV13-04-090** | Male | 67 | 105.5 | 1.68 | 37.38 | FS | 0-24 |
| **OV14-01-081** | Female | 56 | 95 | 1.57 | 38.54 | CS | 25-44 |
| **OV13-04-042** | Male | 54 | 113 | 1.68 | 40.04 | NS | 0 |
| **OV13-04-173** | Male | 52 | 106 | 1.61 | 40.89 | NS | 0 |
| **OV14-01-121** | Male | 59 | 128 | 1.7 | 44.29 | CS | >45 |
